# Supplementary material for: Inverted activity patterns in ventromedial prefrontal cortex during value-guided decision-making in a less-is-more task
Source: Nat Commun. 2017 Dec 1;8:1886. doi: 10.1038/s41467-017-01833-5 (PMC5709383; doi:10.1038/s41467-017-01833-5)
Supplement: Supplementary file 1 — Supplementary Information [file 41467_2017_1833_MOESM1_ESM.pdf]

Inverted activity patterns in ventromedial prefrontal cortex during value-guided decision-making in a less-is-more task

Georgios K. Papageorgiou, Jerome Sallet, Marco K. Wittmann, Bolton K.H. Chau, Urs Schüffelgen, Mark J. Buckley, Matthew F.S. Rushworth

Supplementary Figures

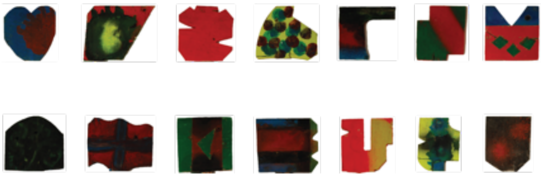

Supplementary Figure 1. Related to Figure 1. Experiment 1: Conditioned stimuli. All 14 stimuli used during experiment 1.

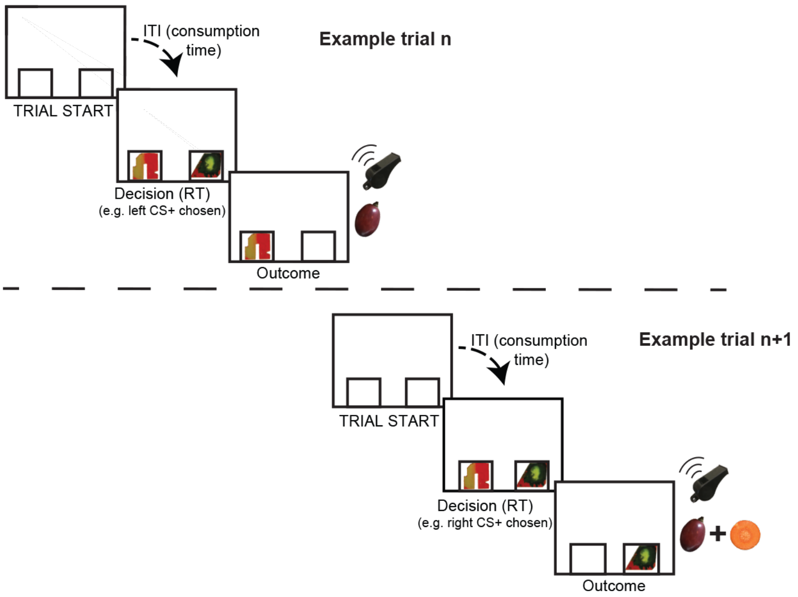

Supplementary Figure 2. Related to Figure 1. Experiment 1: Trial structure. Example trial sequence illustrating two instances of trials involving decisions between the same stimuli (HV associated CS+ chosen in trial n and CV associated CS+ chosen in trial n+1).

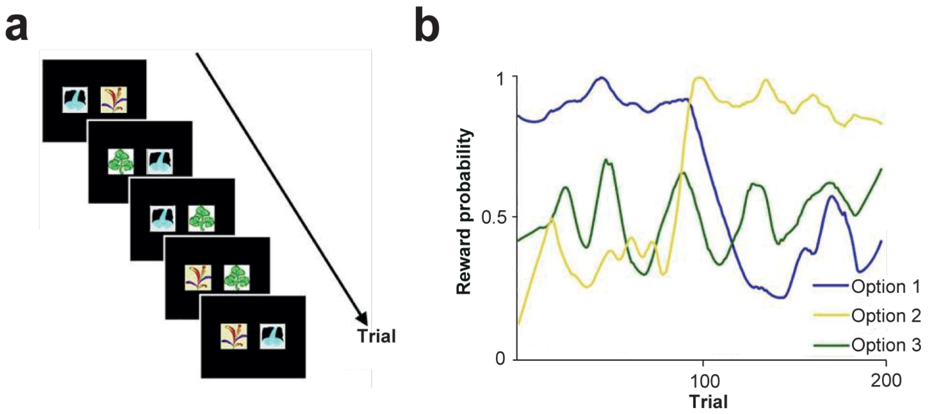

Supplementary Figure 3. Related to Figure 5 and Supplementary Fig. 6. Experiment 3: Trial structure. (a) Three-option probabilistic reward reversal task. On every trial, two out of three options were offered to the animals to choose. **(b)** Each option was associated with a probability of reward, as opposed to being linked in a deterministic manner. The reward probability associations changed over the course of the 200 trials of the experiment[1].

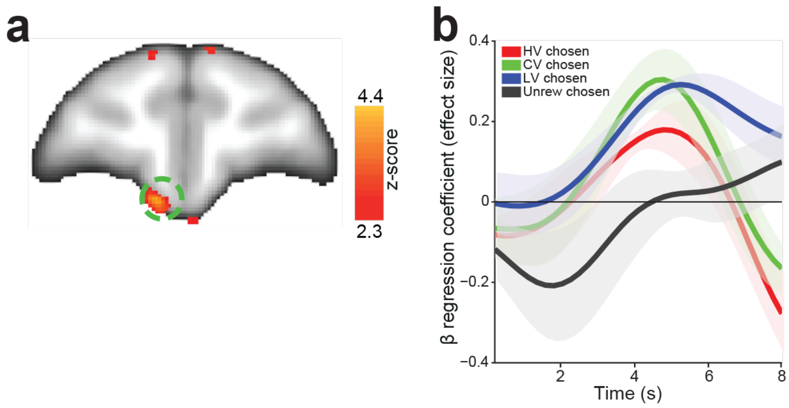

Supplementary Figure 4. Related to Figure 3, 4 and Supplementary Fig. 6. CS value-related activity. (a) Part of the variance in vmPFC activity (4, 15, -1; green circle) simply reflected whether decisions were guided by learned stimulus-reward associations as opposed to trials when animals responded to either the CS- or the blank side of the screen (GLM-2, contrast 2). **(b)** Further analysis of this activity revealed that it was higher when decisions were directed to any reward-associated stimulus as opposed to unrewarded stimuli.

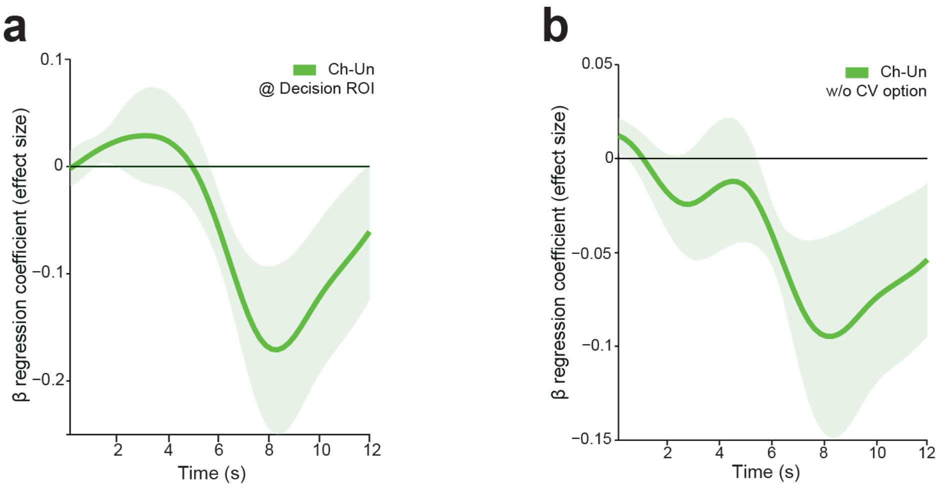

Supplementary Figure 5. Related to Figure 3. Control analyses for "inverse" value activity pattern. (a) Value-related activity at the decision-related ROI (GLM-1) and **(b)** in choices in which the CV option is not available (GLM-2), in experiment 2. **(a)** The difference in value between the chosen and unchosen option values (shown in green color) had a significant impact on vmPFC activity (after square root transformation: t_3=-3.4307, p=0.041) in an ROI around the peak of the main effect of decision-making (Fig. 3a). Such a pattern of activity suggests the main effect of taking a decision (activity that changes whenever a decision is taken regardless of the values of the options considered; shown in Fig. 3a, b) is found in vmPFC in the same regions as activity related to the key variable – the difference in value between the choice taken and the choice rejected – that should drive each decision (shown also in Fig. 3d-f). **(b)** The regression coefficients relating the BOLD signal to the difference between chosen and unchosen options at the time of choice in experiment 2 is plotted in the same manner as in figure 3e but now trials in which the CV option was offered have not been included in the analysis. This means that the chosen-unchosen regression coefficients are based on data from trials on which only the single component HV, LV, and unrewarded options were offered to animals.

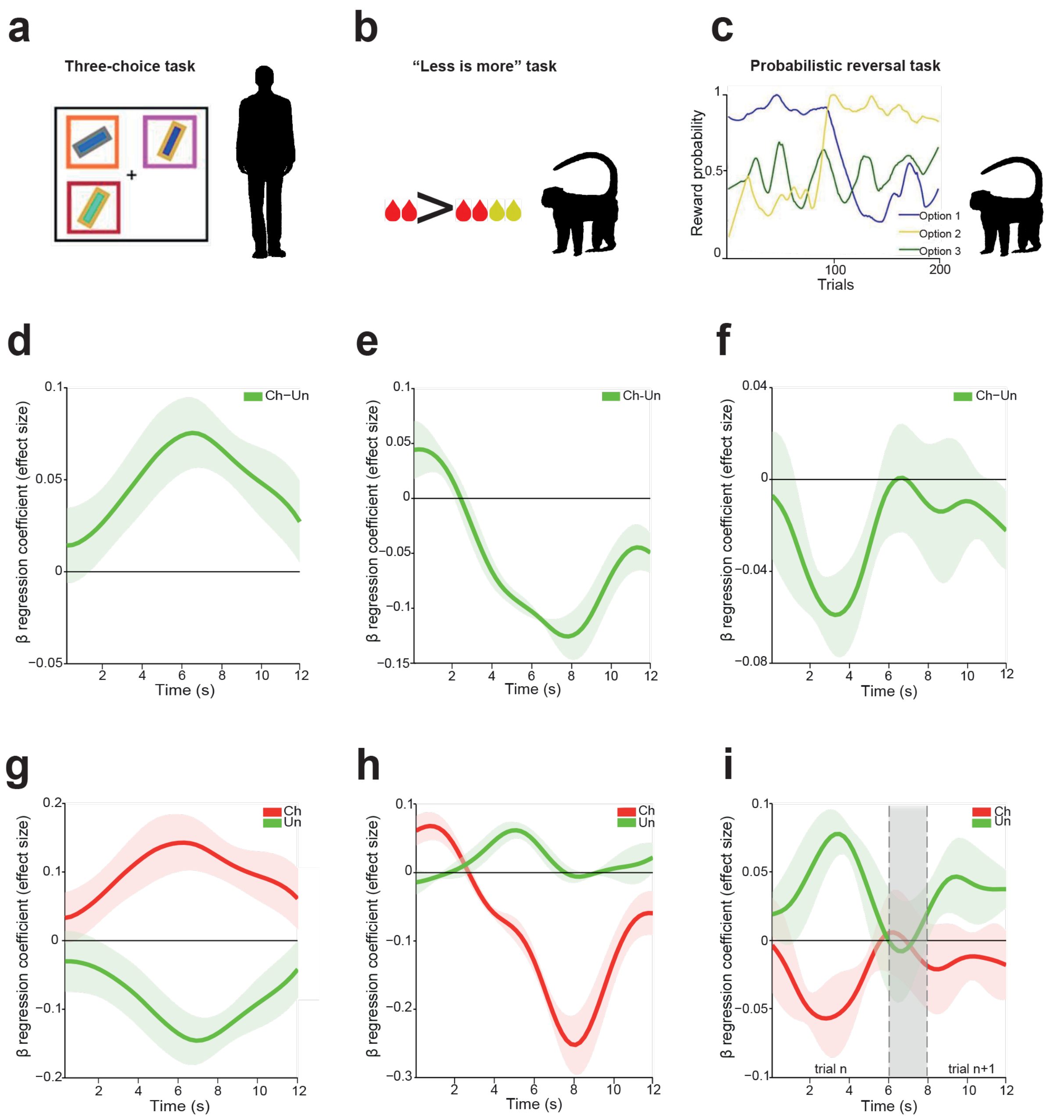

Supplementary Figure 6. Related to Figure 3, 5 and Supplementary Fig. 9. Cross-species comparison during decision-making. Left hand column presents a re-analysis of data recorded from human vmPFC by Chau and colleagues[2] **(a)** while central and right columns show data recorded in the macaque in **(b)** experiment 2 ("less is more" task; see Fig. 3) and **(c)** experiment 3 (three option probabilistic reward reversal task: see Fig. 5), respectively. Panels **(d)**, **(e)**, and **(f)** show vmPFC activity as a function of the difference in value between the chosen and unchosen option in each decision taken in the three experiments. The difficulty of deciding between one choice and another is a function of the difference in their values; when the choices' values are closer together it is more difficult to choose between them and decisions are less consistent. In such situations, the unchosen choice value tends to be higher. In humans vmPFC activity is positively related to the difference in value between the choice taken and the choice rejected **(d)**. By contrast, activity in macaque vmPFC increased in experiments 2 **(e)** and 3 **(f)** when decisions were difficult; it was an inverse function of the difference between the value of the choice taken and the choice rejected. The effects shown in panels d, e, and f are unpacked in panels **(g)**, **(h)**, and **(i)**, respectively. Activity in macaque vmPFC decreases as the value of the chosen option increases and increases as the value of the unchosen option increases in experiment 2 **(h)** and experiment 3 **(i)**. In human vmPFC, in an experiment in which chosen and unchosen values also vary continuously over the course of the experiment as in

experiment 2, it is clear that chosen and unchosen value are associated with positive and negative effects respectively **(g)**. By contrast, in experiment 3 trials were performed quickly so that activity in the first seven seconds, approximately, reflects the current trial (trial n). Later activity reflects decisions on subsequent trials (trial n+1). The grey vertical bar indicates the approximate boundary between trial n and n+1. On 66% of occasions the option chosen on trial n would be offered again on trial n+1 (and it was often chosen again) and on 66% of occasions the unchosen option on trial n would be offered again (in which case it was frequently unchosen again) and so the contrasts for trial n capture activity also on trial n+1.

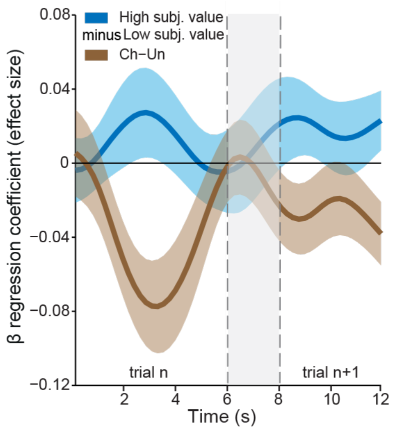

Supplementary Figure 7. vmPFC activity related to the signed difference (ch-un) and absolute difference(|ch-un|) between chosen and unchosen options. The signed difference in between the chosen and unchosen option values (shown in brown color) had a significant impact on vmPFC activity (one-sample t-test: t_3=-3.155, p=0.004) while the absolute difference in the options' values regardless of choice (shown in blue color) did not (one-sample t-test: t_3=0.930, p=0.362). Such a pattern of activity suggests that vmPFC activity is intimately related to the guidance of behavior and/or the current focus of attention.

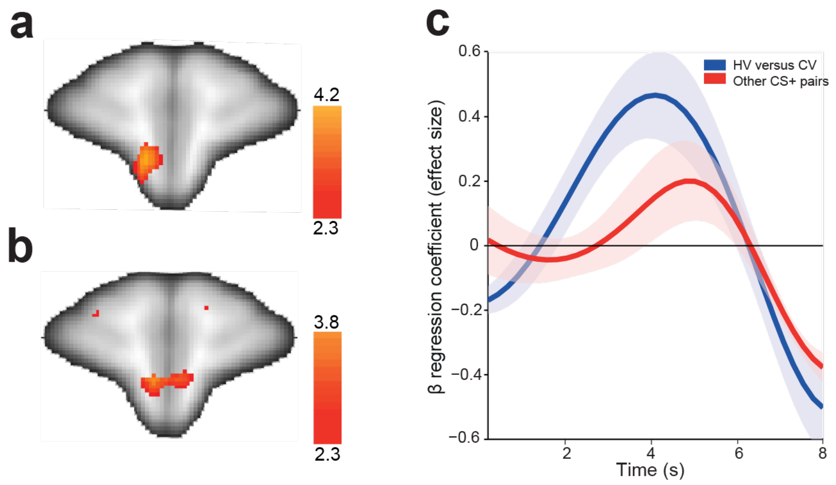

Supplementary Figure 8. (a) Related to Figure 4. Decision-related activity during lesion-affected/unaffected choices. Activity is present throughout vmPFC on HV versus CV trials (GLM-1, contrast 1). **(b)** Activity difference between the HV versus CV trials (which were affected by the lesion) and other trials (which were unaffected by the lesion) (GLM-1, contrast 8). **(c)** ROI time course of effects for illustration.

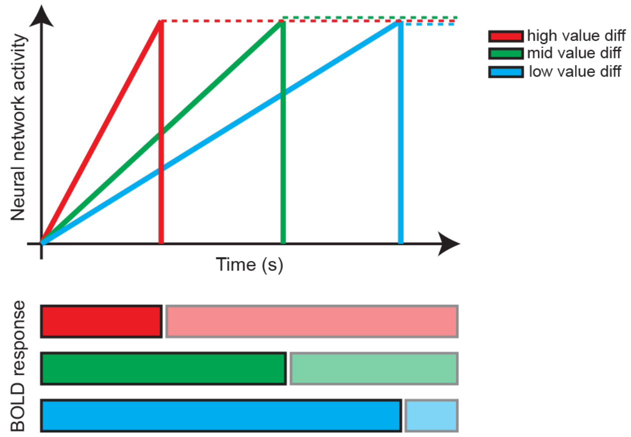

Supplementary Figure 9. Related to Figure 3-5 and Supplementary Fig. 6. Neural network model. (a) Whether activity is positively or negatively related to difficulty may depend on basic features of network[3]. Models such as drift diffusion and biophysical attractor models have shared features and predict which signals should arise in vmPFC and in which order but they do not predict the sign of BOLD changes. If activity in the network is maintained only until decisions are taken then decision difficulty will be associated with increasing BOLD activity but if activity in the network is maintained even after the decision is taken then decision difficulty will be associated with decreasing BOLD activity.

The figure shows activity in a neural network reflecting the relative evidence in favor of the chosen option as opposed to the unchosen option over time. Colors indicate decisions with increasingly close value differences and hence increasing difficulty. When the decision is difficult (low value difference, blue) the network takes longer to move to an attractor state in which a single population is active (point at which the straight lines peak). If network activity decays once the decision is made (approximately vertical red, green, and blue lines in top part of figure) then the integrated index of activity that is reflected in the sluggish BOLD signal is greater when the decision is difficult; left bottom bars indicate expected BOLD response for the decision which predominantly reflects the time-period during which the network moves into an attractor state (difficult decisions, longer integration time, stronger BOLD signal). Such a pattern where value difference is negatively related to the BOLD response is seen in macaques in Fig. 3 and 5 and Supplementary Fig. 6. If, however, the attractor state is maintained even after the decision is taken then, instead of decaying, activity will be as shown by the dashed lines in the top part of the figure. Such a pattern of activity is plausible because biophysical networks of this type have been used to model the maintenance of memory states over short time periods[4]. Now the activity index produced by integration of the sluggish BOLD signal will also reflect the post-decision time. The post-decision period is reached soonest when the decision is easy and choice values are far apart. Now BOLD levels associated with the three decisions are approximated by the length of the shaded bars on the right-hand side of the bottom part of the figure. Easy decisions are now associated with the greatest aggregate activity. Such a pattern is seen in humans in Supplementary Fig. 6.

Alternatively, the initial, pre-decisional network activity may be higher in macaques compared to humans and hence one pool of neurons may need to be suppressed until an attractor state is reached. Such a model would also predict a negative value difference signal and is also consistent with our observation that the main effect of taking a decision is positive in monkeys but negative in humans (Fig. 3a). Wong and Wang[5] have described how changes in a single network parameter, the level of recurrent excitation, can determine whether or not the network can make a decision and, if it can make a decision, whether the representation of the choice is maintained in a high firing attractor state. Such a simple change could determine whether the aggregate activity recorded from such a network resembled the pattern seen in humans or in macaques (Supplementary Fig. 9). It is quite plausible that simple features of neurons in networks might vary across species.

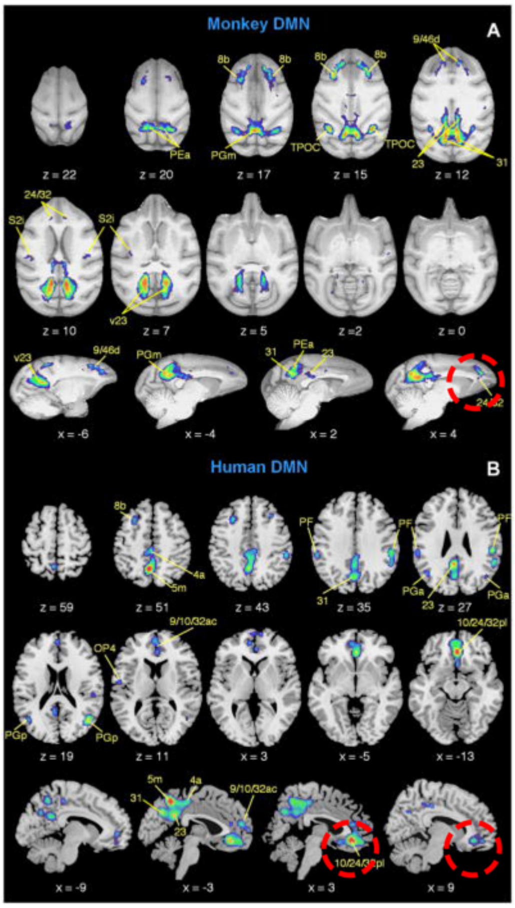

Supplementary Figure 10. Related to Figure 3. Resting State networks in humans and macaques. The "default mode network (DMN)", "resting state", or "task negative" fMRI-recorded activity pattern in macaque macaques (top) and humans (bottom) as defined by Mantini and colleagues[6]. The networks are similar in both species at many points but there are differences in medial frontal cortex. Default mode type activity is found in the human vmPFC and adjacent perigenual anterior cingulate cortex (pgACC) labelled 10/24/32pl (bottom right two panels highlighted with a red circle). In monkeys, default mode type activity is found in a relatively dorsal part of ACC, labelled 24/32 (bottom right panel in macaque results highlighted with a red circle) but not in vmPFC. While other studies have reported default mode-type activity in many of the areas of the macaque brain highlighted by Mantini and colleagues, like Mantini and colleagues, they also failed to find default mode-like activity in vmPFC[7,8]. Figure based on study by Mantini and colleagues[6] and used with permission.

Supplementary Data Tables

S1 **Learning phase**	S2 **Revision sessions**	S3 **Main task**	S4 **Revision sessions**	S5 **Main task**	S6 **Learning phase**
**Set A**	**Set A**	**Set A**	**Set A**	**Set A**	**Set B**
1-4 days	*1-2 days*	*2 days*	*1-2 days*	*1 day*	*1-4 days*
S7 **Revision sessions**	S8 **Main task**	S9 **Revision sessions**	S10 **Main task**	S11 **Revision sessions**	S12 **Devaluation phase**
**Set B**	**Set B**	**Set A**	**Set A&B**	**Set A|B**	**Set A|B**
1-2 days	*3 days*	*1-2 days*	*3 days*	*1-2 days*	*1 day*

Supplementary Table 1. Related to Figure 1. Experimental timeline. From S1-S12 the order of the different stages of the experiment is presented.

**GLM-1 regressors**	**GLM-2 regressors**
Cue Onset events	
HV versus Blank side	CS+ Chosen
CV versus Blank side	Parametric: Chosen + Unchosen value
LV versus Blank side	Parametric: Chosen – Unchosen value
HV versus CS-	CS-/Blank side Chosen
CV versus CS-	
LV versus CS-	
HV versus CV	
HV versus LV	
CV versus LV	
Discarded trials (i.e. choices with RT > 1SD)	
Reward delivery events	
HV versus Blank side	CS+ Chosen
CV versus Blank side	CS-/Blank side Chosen
LV versus Blank side	
HV versus CS-	
CV versus CS-	
LV versus CS-	
HV versus CV	
HV versus LV	
CV versus LV	
Discarded trials (i.e. choices with RT > 1SD)	
Response events	
Left Hand	Left Hand
Right Hand	Right Hand
Left Hand without HRF	Left Hand without HRF
Right Hand without HRF	Right Hand without HRF

Supplementary Table 2. Related to Figure 2-4 and Supplementary Fig. 4-7. Regressors in GLM-1 and GLM-2

Based on GLM-1 and GLM-2 the following contrasts of regressors were constructed:

GLM-1 contrasts	GLM-2 contrasts
Cue Onset time	
1. Decisions between HV and CV (Fig. 4a)<br>2. Decisions between HV and LV (Fig. 4a)<br>3. Decisions between HV and an unrewarded option (CS- or blank side of screen) (Fig. 4a)<br><br>4. Decisions between CV and LV (Fig. 4b)<br>5. Decisions between CV and an unrewarded option (CS- or blank side of screen) (Fig. 4b)<br><br>6. Decisions between LV and an unrewarded option (CS- or blank side of screen) (Fig. 4c)<br><br>7. Lesion-unaffected decisions = HV versus LV + CV versus LV<br><br>8. HV versus CV – Lesion-unaffected decisions (this contrast compares decisions that were affected by the lesion and decisions that were not affected by the lesion; Supplementary Fig. 7b, c)	1. Decision = CS+ Chosen + CS-/Blank side Chosen (Fig. 3a, b)<br><br>2. Decisions guided by stimulus value = CS+ Chosen – CS-/Blank side Chosen (Supplementary Fig. 6)<br><br>3. Chosen-unchosen value difference (Fig. 3d-g, Supplementary Fig. 5)

Supplementary Table 3. Related to Figure 2-4 and Supplementary Fig. 4-7. Contrasts in GLM-1 and GLM-2

Event	Brain regions	MNI coordinates			z-score
Cue Onset		x	y	z	
	vmPFC/mOFC	4	21	2.5	4.26
z>2.3	mOFC	7	19.5	7.5	5.55
p<0.05	lOFC	19	9	-4.5	5.08
	SMG	26.5	1	1.5	4.95

Supplementary Table 4. Related to Figure 3. Activity at time of decision (GLM-2)

Event	Brain regions	MNI coordinates			z-score
Cue Onset		x	y	z	
z>2.3	mOFC	-8.5	16.5	9.5	-4.29
p<0.05	mOFC	7.5	14.5	9.5	-3.84
	pgACC	-1	23.5	10	-3.77
	MFG	14.5	5.5	16	3.35
	Cerebellum	-12.5	-28	-19.5	4

Supplementary Table 5. Related to Figure 3, Supplementary Fig. 4 and Supplementary Fig. 7. Activity related to decision variable: chosen value-unchosen value (GLM-2)

Supplementary References

1. Chau, B. K. H. *et al.* Contrasting Roles for Orbitofrontal Cortex and Amygdala in Credit Assignment and Learning in Macaques. *Neuron* **87,** 1106–1118 (2015).
2. Chau, B. K. H., Kolling, N., Hunt, L. T., Walton, M. E. & Rushworth, M. F. S. A neural mechanism underlying failure of optimal choice with multiple alternatives. *Nat. Neurosci.* **17,** 463–470 (2014).
3. Kolling, N. *et al.* Value, search, persistence and model updating in anterior cingulate cortex. *Nat. Neurosci.* **19,** 1280–1285 (2016).
4. Wang, X.-J. Probabilistic decision making by slow reverberation in cortical circuits. *Neuron* **36,** 955–968 (2002).
5. Wong, K.-F. & Wang, X.-J. A recurrent network mechanism of time integration in perceptual decisions. *J. Neurosci.* **26,** 1314–1328 (2006).
6. Mantini, D. *et al.* Default mode of brain function in monkeys. *J. Neurosci.* **31,** 12954–12962 (2011).
7. Mars, R. B. *et al.* On the relationship between the ‘default mode network’ and the ‘social brain’. *Front. Hum. Neurosci.* **6,** 189 (2012).
8. Vincent, J. L. *et al.* Intrinsic functional architecture in the anaesthetized monkey brain. *Nature* **447,** 83–86 (2007).
